# Scraps

**Published:** 1897-09

**Authors:** 


### SCRAPS

PICKED UP BY THE ASSISTANT-EDITOR.

**Clinical Records** (20).—A hedge doctor (a quack) in Ireland was being examined at an inquest on his treatment of a patient who had died. "I gave him ipecacuanha," he said. "You might just as well have given him the *aurora borealis*," said the Coroner. "Indade, yer honor, and that's just what I should have given him next, if he hadn't died."—*St. Thomas's Hospital Gazette.*

**Mr. Hall Caine on Medical Matters.**—In Mr. Hall Caine's last novel, "The Christian," some comical perversions of medical knowledge occur. For example, the weak-minded but beautiful Polly Love is killed off by a dose of "half a grain of liquor strychnine." This is, perhaps, not quite in accordance with modern pharmacology, but Mr. Caine unfortunately omits to mention anything about the chemist who weighed out the half grain of the liquid poison. In a medico-legal matter, again, Mr. Caine has established a record, and it is evident that we are quite wrong as to the definition of a still-born child, if this record is to be maintained. In "The Christian" the author describes a still-born child as "one that has breathed but never cried."—*Medical Press and Circular.*

**Longevity of Physicians.**—One of the most curious statistical records that has been compiled this century is that by Dr. Salzmann, of Essling, Wurtemburg, on the average duration of life among physicians. He found, in going over the ancient records of the kingdom, that in the sixteenth century the average duration of life among the class was 36.5 years; in the seventeenth century, 45.8; in the eighteenth, 49.8, and at the present time they reach the favourable average of 56.7. It appears from the foot-notes to the above that this very great increase in longevity is due to the disappearance of the "black pest," the introduction of vaccination, and the great diminution in the number of typhus epidemics, three classes of diseases which formerly decimated the medical practitioners.—*Church Family Newspaper.*

**Medical Missions.**—As the Indian Frontier is now much in our minds, it is of special interest to read in the last Report of the Church Missionary Society, in a note on the recent death of the Rev. John Williams, the native pastor and medical missionary of Tank, that by his gentle and winning manners, his kindness to the people, and his medical skill, he won his way among the Waziri clans, and he was probably the only Christian man in India who could in those days travel unarmed, and without any escort, uninjured throughout the length and breadth of that mountainous country of wild Mohammedans. The Government repeatedly bore witness to the influence which John Williams had gained over these wild tribes, and to the political advantages which they had received through his means. When the Waziris attacked and burnt Tank in 1879, they placed a sentry of their own over the Christian hospital, and over the house of their Christian friend and teacher, from whom they had often heard of the Gospel of Christ, and thus ensured his safety in perilous times.

**Le Choléra en 1831.**—On relira peut-être avec intérêt, en tous cas certainement avec un souvenir ému, les beaux vers que le célèbre et courageux Barthélemy, le journaliste-poète, consacrait au choléra morbus dans son inimitable journal en vers, *Némésis*, le 28 août, 1831.

Où va-t-il ce géant que le monde redoute?
Qui connaît le secret de sa carte de route?
Errera-t-il longtemps sur les cercles germains?
O terreur! De ce globe il sait tous les chemins!
. . . . Né sur le sol heureux qu'embaume l'aromate
Il s'ébat volontiers, dans ses horribles jeux,
Au bord des lacs infects et des marais fangeux.
. . . . Le choléra jaloux, dans son brûlant passage,
D'une teinte verdâtre empreint son frais visage,
Il glace ses pieds nus, brûle ses intestins,
. . . . Suspend des longs baisers la nocturne harmonie,
Et change un cri d'amour en râle d'agonie!

Relisant l'œuvre du fécond écrivain, nous n'avons pu résister au plaiser de recopier quelques vers du journal qui fit tant de bruit à l'époque de l'apparition du choléra en Europe.—*Progrès médical.*

**Medical Philology** (XXIII).—The *Catholicon* gives "Coriandre; *coriandrum*." Mr. Herrtage's note says that "Alexander Neckham, *De Naturis Rerum*, p. 476, assigns the following virtues to Coriander—

'*Et triduana febris eget auxilio coriandri,*
*Et gemini testes dum tumor ambit eos.*
*Lumbricos pellit, tineas delet, sacer ignis,*
*Quam pestem metuit Gallia, cedit ei.*'"

Turner in 1551 says in his *Herbal* that "Coriandre layd to wyth breade or barly mele is good for saynt Antonyes fyre" (*i.e.* erysipelas).

The word comes to us through the Latin from the Greek *κορίαννον*, which is said to be connected with *κόρις*, a bedbug, on account of the peculiar smell of the leaves. The presence of the *d* is an instance of the insertion of an excrescent letter during the consonantal changes which have taken place in words. Skeat gives several native instances of *d* being excrescent after *n* (*Primer of English Etymology*, 1892, p. 94). Other forms of the word were cellendre and coliander, and its variants. In the 1382 Wyclif Bible in Exodus xvi. 31, is "coliaundre," and in the 1388 version "coriandre." Turner in 1538 speaks of "coryander aut colander."

**Medical Examinations.**—The *Post-Graduate*, referring to an examination not for admission to the House Staff of a New York Post-Graduate Hospital, says that there were a few glaring exceptions to the general proficiency which are amusing if not instructive. One person made a classification "infectious, cutaneous, and subcutaneous diseases." Another described scabies as "A disease of the skin due to the presence of a small *germicide*." Still another said, "A person to be vaccinated has the epidermis or skin or epithelium scraped from his body." To the question, What is *trachoma?* was answered: 1st, "Swelling of the *trachea*, causing pressure." 2nd, "Trachoma is a parasite introduced into the human system through food, mainly flesh of hogs." 3rd, "Trachoma is a severe disease of the eyeball, which may terminate in blindness." To the question, What is the *habitat* of tinea tonsurans? was answered, "Habitat, small intestines, diagnosis is made by examination of the stools." 2nd, "Tinea tonsurans—constipation, restlessness, picking of the lips, convulsions—worm may be passed." In tinea circinata another said, "They have itching of anus, scratching—anus red, and may be able to see the worm externally." To the question, What is the *habitat* of each variety of pediculus? the answer was, "Usually a person very lax in the care of his head and body." The following was the entire clinical history given of whooping-cough: "Patient had *diffused* eyes, often blue rings around, when spasms are strong the patient whoops, has asthmatic attacks." Here is an interesting specimen of natural philosophy in reply to a question about ventilation: "Fresh air is generally much cooler than foul air, and in consequence rushes through ventilators, windows, doors, etc., driving the warm air before it."

**A Roman Doctor.**—In the *Spectator* of August 28th, 1897, A. C. has the following "after Ausonius":

Wise Arruns, asked "How long will Caius live?"
Replied, "Three days the fatal sisters give;"
And Arruns knew the prophet's art. But lo!
Stronger than gods above or gods below,
Euschemon comes; his healing arts he tries,
And in a single day poor Caius dies.

This, I imagine, must be a paraphrase of the 73rd Epigram, which in the Delphin Classics edition of 1730 is thus printed:

Languenti Marco dixit Diodorus haruspex
  Ad vitam non plus sex superesse dies.
Sed medicus divis fatisque potentior Alcon,
  Falsum convicit illico haruspicium.
Tractavitque manum victuri, ni tetigisset.
  Illico nam Marco sex periêre dies.

This was not the only mark of attention which Alcon received from Ausonius, who in the next epigram writes:

Alcon hesterno signum Jovis attigit. Ille,
  Quamvis marmoreus, vim patitur medici.
Ecce hodie jussus transferri ex æde vetustâ,
  Effertur: quamvis sit Deus, atque lapis.

Perhaps some reader of the *Journal* will kindly send me a Greek or English version of this.

The lot of the doctor among the ancient Romans was not a very desirable one. The lampooning by smart and cheap epigrammatists was only a small portion of his trouble, for in addition to being domineered over by the haughty patrician, he was sometimes compelled to perform unnecessary operations upon the slaves, and if a successful practitioner he even ran the risk of being poisoned by his envious rivals.

**Obstetrics, Home and Foreign** (4).—" From an ancient 4to. MS, formerly in the collection of Herbert, dated 1475, I transcribe the following charm, or more properly charect, to be bound to the thigh of a lying-in woman: 'For woman that travelyth of chylde, bynd thys wryt to her thye: In nomine Patris ✠ et Filii ✠ et Spiritus Sancti ✠ Amen. ✠ Per virtutem Domini sint medicina mei pia crux et passio Christi. ✠. Vulnera quinque Domini sint medicina mei. ✠. Sancta Maria peperit Christum. ✠. Sancta Anna peperit Mariam. ✠. Sancta Elizabet peperit Johannem. ✠. Sancta Cecilia peperit Remigium. ✠. Arepo tenet opera rotas. ✠. Christus vincit. ✠. Christus regnat. ✠. Christus dixit Lazare veni foras. ✠. Christus imperat. ✠. Christus te vocat. ✠ Mundus te gaudet. ✠. Lex te desiderat. ✠ Deus ultionum Dominus. ✠. Deus preliorum Dominus libera famulam tuam N. ✠ Dextra Domini fecit virtutem. a. g. l. a. ✠ Alpha ✠ et Ω. ✠. Anna peperit Mariam, ✠ Elizabet precursorem, ✠ Maria Dominum nostrum Jesum Christum, sine dolore et tristicia. O infans sive vivus sive mortuus exi foras ✠ Christus te vocat ad lucem. ✠. Agyos. ✠ Agyos. ✠ Agyos. ✠ Christus vincit. ✠ Christus imperat. ✠ Christus regnat. ✠ Sanctus ✠ Sanctus ✠ Sanctus ✠ Dominus Deus. ✠ Christus qui es, qui eras, ✠ et qui venturus es. ✠ Amen. bhurnon ✠ blictaono ✠ Christus Nazarenus ✠ Rex Judeorum fili Dei ✠ miserere mei ✠ Amen.' "—Brand's *Popular Antiquities*, ed. Bohn, 1849, vol. ii., pp. 67-8.

I shall be glad to have an explanation of "Arepo tenet opera rotas," "a. g. l. a.," "bhurnon," "blictaono."

" The description of an Indian labor, as given me by Dr. McCoy from his experience at the Nisqually agency, will give an excellent idea of the assistance which is tendered the Indian woman in her confinement. 'The midwives, of whom there are two in attendance, call upon the Great Spirit for help in a muttering tone, and in the same tones name over the parts immediately connected with the parturient effort, and often all the joints and limbs of the body. By applying their hands to the abdominal walls they try to ascertain the position of the fetus *in utero* and usually to correct malpresentation. They use oil to anoint the parts, and just before the expulsion of the child give medicines to increase the pains.' "—Engelmann's *Labor among Primitive Peoples*, 1882, p. 133.

**Aphorisms** *(A long way after Hippocrates):*

'Ο βίος βραχὺς, ἡ τέχνη μακρὴ, ὁ καιρὸς ὀξὺς,
ἡ πεῖρα σφαλερὴ, ἡ κρίσις χαλεπή.

1. Life is short, the curriculum long; examiners treacherous, tips fallacious; fees exorbitant, diplomas valueless.

2. Of making many treatises there is no end, and a superfluity of lectures causeth ischial bursitis.

3. Whoso seeketh appointments seeketh grief and heartburning, for the thyrsis-bearers* are many, but the inspired few.

4. The art consists in three things—the disease, the patient, and the house-physician.

5. He that would acquire a competent knowledge of medicine ought to be possessed of the following advantages:—Natural ability; competent instructors; favourable conditions for study; early tuition; indomitable perseverance; ample capital.

6. General practice is harasssing; specialism lucrative; midwifery laborious; life assurance responsible; hospital measures radical.

7. Dentistry is golden; ophthalmology cleanly; throat-work sanguinary; otology circumscribed; pessary practice debasing; hygiene elevating; clubs demoralising.

8. Whoever would prosper in private practice must first master all these; must possess presence of mind, gentlemanly manners, true sympathy, an iron constitution, an even temper, a head for figures, knowledge of human nature, a devoted wife.

9. The successful practitioner understands three things—the digestion of infants, the nervous organisation of women, the infirmities of age.

* A term applied to the general body of worshippers in a Greek [Bacchic] temple.

10. The physician is a frequent worshipper in the temple of the dead, but the surgeon's memory for mortal cases is ephemeral.

11. The physician recognises the limits of his art, but the surgeon discredits himself by attempting the impossible.

12. A scientific operation requires three conditions—accurate diagnosis, carefully-planned details, patient nursing.

13. The most acute cases are those which are suitable for the surgical wards; the next most acute are suitable for the septic wards; after these come cases suitable for clinical; then those suitable for the medical wards; cases more chronic than these are cases suitable for another hospital; and if there be any others yet more chronic than these, they are eminently fitted for a parish infirmary.—*Guy's Hospital Gazette.*

**Medical "Bits of Old Bristol"** (4).—So far back as 18 Edward III., 1344-5, the municipal authorities made some effort to prevent the spread of communicable disease. In that year the Commonalty of Bristol revised their ordinances and by-laws, which were codified by William de Colford, who was then Recorder. Barrett (*Hist. of Bristol*, p. 675) says: "Amongst many regulations then made it was ordered that no leprous man stay within the precincts of the town, nor any common woman remain within its walls; and if such women be found residing there, then the doors and windows of the houses shall be unhung and carried by the serjeants of the mayor to the house of the constable of the ward, and there to be kept till the women be removed.—That no whore should ever appear in the streets, or even within the bars in St. James's without their head covered (capite stragulato)."

In the *Journal* for December, 1896, I gave some information about the local accommodation for lepers. Through the courtesy of Mr. Alfred E. Hudd, who has referred me to a very interesting paper by the Rev. C. S. Taylor in Part I. of Vol. III. of the *Proceedings of the Clifton Antiquarian Club*, I am able to add to the information I then gave about St. Laurence's Hospital. In 1892 Mr. Taylor's attention was called to a lease of a house in "Weste strete," which in 1438 the Master or Warden of the Hospital granted to Thomas and Elena Palmer and their son William. Mr. Taylor cites William Wyrcestre's description of the position of the Hospital. Wyrcestre says (*Itinerarium*, ed. 1778, p. 210): "Viæ longitudo ab exteriori parte Laffordys-yate, continuando usque ad ecclesiam et domum ac hospitale pertinenti dictæ ecclesiæ, continet in longitudine in comitatu Gloucestriæ incipiente, et versus viagium ad civitatem Londoniarum transeuncium per forestam de Kyngyswod, incipiente apud Laffordys-yate continet usque dictum hospitalem Sancti Laurencii per . . . fundatam, quamvis modo pertinet tempore Edwardi regis quarti collegio et ecclesiæ de Westbery, 1200 gressus per W. Worcestre mensuratis."

Accepting Archdeacon Norris's estimate of Wyrcestre's gressus as two feet, Mr. Taylor concludes that the hospital occupied a site "rather nearer to Lawford's Gate than the existing Church of St. Lawrence, and on the same side of the road," and says "there could not have been a better site than the one which Prince John chose for the home of the Bristol lepers. At a sufficient distance, about half-a-mile, from the City gate, yet not so far off that the citizens would forget the needs of their unhappy brethren; in the neighbourhood of the forest of Kingswood, where there would be few inhabitants, and where the fresh wind from the forest would purify, as far as possible, the tainted precincts. There, in peace, the inmates would die their living death, knowing that at any rate all that could be done for them had been done." Mr. Taylor adds that "in a Map of Kingswood Forest which belongs to Mr. Chester Master, and which is dated 1610, the Hospital is represented on the North side of the road at about the point at which it is joined by Leadhouse Lane."

In their order about the common women the authorities may have been actuated by a desire to improve the public health as well as public appearances. There was nothing unusual in constituted authority concerning itself with such matters. The Stews in Southwark, a row of 18 houses set apart as brothels, were under the jurisdiction of the Bishop of Winchester. Many writers referred to this. Shakspere and Ben Jonson have indirect allusions to it (1 *Henry VI.*, I. iii. 53; *Troilus and Cressida*, V. x. 55: *An Execration upon Vulcan*). In these passages they used the term employed to describe one of the physical evils resulting from a visit to the Stews, and which chronicled the episcopal connection with them. In Florio's 1598 Italian and English Dictionary known as *A Worlde of Wordes*, we find "Pannochia, *a winchester goose, an impostume about the priuie members*," and "Pudendágra, *a disease about the priuie parts like a winchester goose.*" Cotgrave, in 1611, gives "A botch in the Groyne, or yard; a winchester goose," as the equivalent of the French *clapoir*, and he has much the same definition of *poulain*. Regulations for the management of these houses had been made by Parliament in 8 Henry II., 1161–2. Amongst other things it was enacted that "No Stew-holder should keep open his doors on the Holy-daies, or keep any in his house on those daies, or receive any man's wife, or any woman of religion." (Stow's *Survey of London*, ed. 1633, pp. 448-9, and Fuller's *Church History*, ed. 1655, pp. 240-1), When it was decreed that "No Stew-holder [was] to keepe any woman, that hath the perillous infirmity of Burning," it is clear that even in the twelfth century the guardians of the public health saw the necessity of something like a Contagious Diseases Act. An interesting dissertation on Burning or Brenning—an early term for gonorrhœa—appears in vol. xxx. of the *Philosophical Transactions* (*Abridged edition*, vol. vi., 368–73). The legalised existence of the Southwark houses was too much for the moral Henry VIII., who disestablished them in 1546.

The mention, in the Bristol order, of the district described as "within the bars in St. James's" is curious, as that was beyond the jurisdiction of the town. Towards St. James's the course of the Frome was at that time the line of defence of the city, and in this part of it there were no bars in the sense of barriers "closing the entrance into a city, formed originally of 'posts, rails, and a chain,' afterwards applied to the gate by which these were replaced." The expression "within the bars in St. James's" seems to imply that, in the area under the jurisdiction of the Priory of St. James, there was an enclosed space to which these women were limited, and the statement in Colford's code need not be taken to mean that the city had authority over the district, but merely to record that such a regulation existed in the immediate neighbourhood. A considerable population had grown up in this suburb of Bristol, in which the Priory was a large and conspicuous feature, covering the ground from Marlborough Street to Broadmead and from Lower Maudlin Street to North Street. "The memory of the prioral grange survives in the name of St. James's Barton." I offer the foregoing suggestions with diffidence, and I shall be glad to be set right if I have made any mistake about the limits of the civic authority at the date in question.

Mr. John Latimer tells me that he has seen "numerous early leases in which the Barrs are mentioned, but there is never any explanation as to what the Barrs really were. The property is described as 'juxta le Barres' or 'apud le Barres,' and that is all."

The neighbourhood had for a long time an evil reputation. In 1440 William Wyrcestre describes it as "lez barrys ubi mulieres fatuæ morantur." (*Op. cit.*, p. 214); and elsewhere, in the same work (pp. 168, 255), he has something to say about the people of the district, the position of which was practically that of the present Barrs Street. In the Wardens' accounts for 1672 there is an item of "Paid to Buck for hunting the whores out of the parish, 6d." (*Bristol: Past and Present*, 1881, vol. ii., p. 40).

Writing in the end of the sixteenth century on the question of the public stews, Camden (*Britannia*, ed. 1789, vol. ii., p. 9) approves the abolition of these houses kept up "under the specious pretence of allowance for human infirmity"; but his editor, Gough, thinks "it is still a question whether the licensing such places by authority would not tend more to preserve the morals as well as the health of the subjects than the present unrestrained and promiscuous indulgence" (*Op. cit.*, p. 27). The arguments for and against the existence of such houses are well put by Fuller (*loc. cit.*), who sums up the matter by saying that licensing them added a national sin to the personal sin committed by those who resorted to them.

